# De-epithelialized Free Gingival Graft for Gingival Augmentation: A Case Report

**DOI:** 10.7759/cureus.107511

**Published:** 2026-04-21

**Authors:** Shivani G, Rekha R Koduganti, Veerendranath Reddy Panthula, Swetha Manchala, S Bhavana

**Affiliations:** 1 Department of Periodontics, Panineeya Institute of Dental Sciences and Research Centre, Hyderabad, IND

**Keywords:** dentinal hypersensitivity, free gingival graft, gingival recession, laser de-epithelialized free gingival graft, recession coverage

## Abstract

Gingival recession is a commonly encountered mucogingival condition that may lead to root exposure, dentinal hypersensitivity, and compromised aesthetics. Various surgical techniques have been proposed for root coverage, among which the de-epithelialized free gingival graft (DFGG) combines the advantages of both free gingival grafts (FGGs) and connective tissue grafts. A 26-year-old male patient presented with gingival recession in relation to mandibular central incisors (31 and 41). An FGG was harvested from the palatal donor site using a scalpel and subsequently de-epithelialized extraorally using a laser to obtain a connective tissue graft. The graft was placed at the recipient site and stabilized with sutures. Healing was uneventful, and a significant reduction in gingival recession was observed, along with an increase in the width of keratinized gingiva and improved aesthetic outcome. Laser-assisted DFGG appears to be a predictable and effective technique for the treatment of gingival recession, providing favorable clinical and aesthetic outcomes.

## Introduction

Gingival recession is defined as the apical migration of the gingival margin beyond the cementoenamel junction, resulting in exposure of the root surface [1]. It is frequently associated with aesthetic concerns, dentinal hypersensitivity, root caries, and non-carious cervical lesions. The etiology is multifactorial, including traumatic tooth brushing, periodontal inflammation, thin gingival phenotype, mal-positioned teeth, and inadequate width of attached gingiva. Numerous surgical techniques have been developed for root coverage [2]. The free gingival graft (FGG), typically harvested from the palate, is effective in increasing the width of keratinized gingiva but often results in poor aesthetic outcomes. The subepithelial connective tissue graft (SCTG) is considered the gold standard due to its superior aesthetic integration and predictability; however, it is technique-sensitive. De-epithelialized FGG (DFGG) is a modification of the FGG in which the epithelial layer is removed to obtain connective tissue, thereby combining the advantages of FGG and SCTG. Recently, lasers have been increasingly utilized in periodontal therapy due to their ability to provide precise tissue ablation, improved hemostasis, and enhanced wound healing. The present case aims to evaluate the clinical effectiveness of a laser-assisted DFGG harvested from the palate in the management of gingival recession. The rationale for choosing this technique was to harvest sufficient soft tissue from the donor site easily, and its significance can be appreciated in the satisfactory postoperative outcome three months later.

## Case presentation

**Figure 1:**
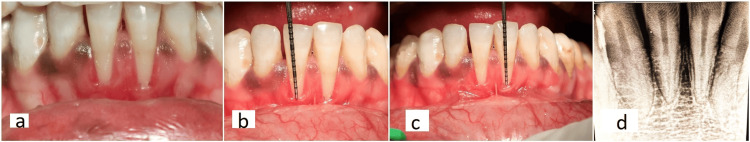
a: Preoperative picture of gingival recession in 31 and 41; b: Measurement of recession height at the baseline with the UNC 15 probe in 41; c: Measurement of recession height at baseline with the UNC 15 probe in 31; d: Preoperative RVG of 31 and 41 UNC: University of North Carolina; RVG: radiovisiography

A 26-year-old male patient reported with the chief complaint of receding gums and sensitivity in the lower anterior region. The patient’s medical history was non-contributory. A clinical examination revealed gingival recession in relation to mandibular central incisors (31 and 41), classified as Miller’s Class IV. The recession depth measured approximately 5 mm in relation to 31 and 5 mm in relation to 41, with inadequate width of keratinized gingiva and a thin gingival phenotype (Figures 1a-1d).

**Figure 2:**
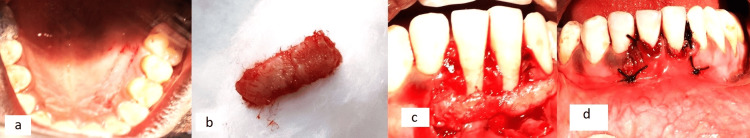
a: Outline of the graft to be harvested from the palate marked; b: The harvested free gingival graft; c: Placement of the graft after laser de-epithelialization in the recipient site; d: Suturing of the flap done

Phase I therapy (scaling and root planing) with oral hygiene instructions was given. Following re-evaluation, surgical intervention was planned. Under local anesthesia, a partial-thickness flap was raised at the recipient site, and thorough root planing was carried out. An FGG was harvested from the palate (donor site) using a scalpel. The harvested graft was subsequently de-epithelialized extra-orally using a diode laser (910nm) to obtain a connective tissue graft. The graft was adapted over the recipient site and stabilized using interrupted sutures (Figures 2a-2d).

**Figure 3:**
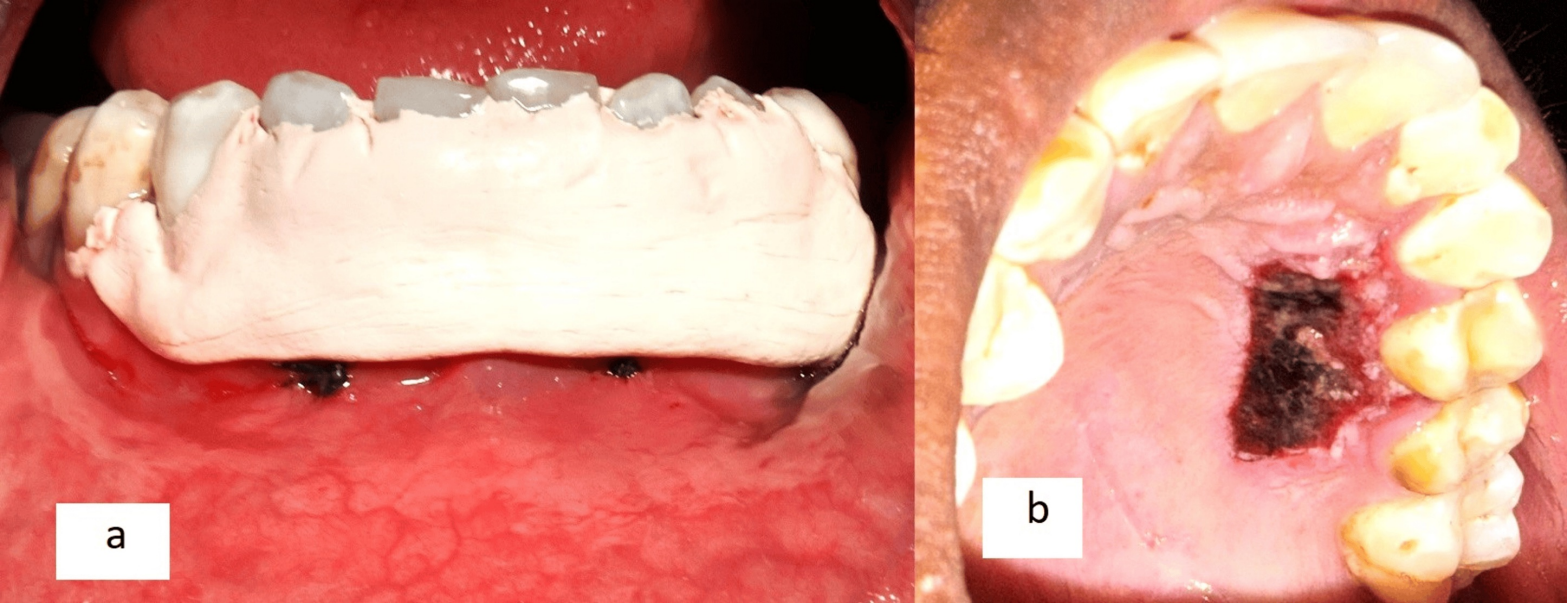
a: Periodontal dressing placed; b: Donor site at one week postoperatively

A periodontal dressing was placed, and postoperative instructions were given. The patient was prescribed an antibiotic and analgesic and advised to use 0.12% chlorhexidine mouth rinse. Healing was uneventful, with satisfactory graft integration and donor site healing after one week (Figures 3a-3b).

**Figure 4:**
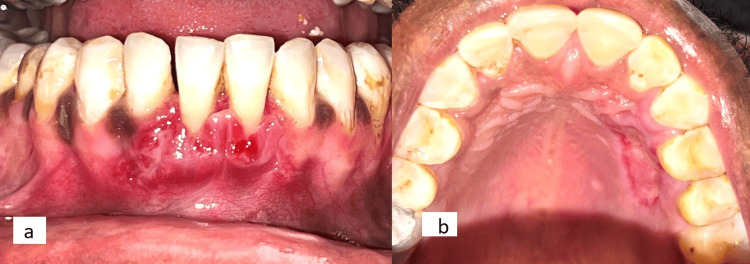
a: Healing at the recipient site at three weeks postoperatively; b: Healing of the donor site at three weeks postoperatively

At three weeks' follow-up, the healing was better appreciated at the donor and recipient sites (Figures 4a-4b).

**Figure 5:**
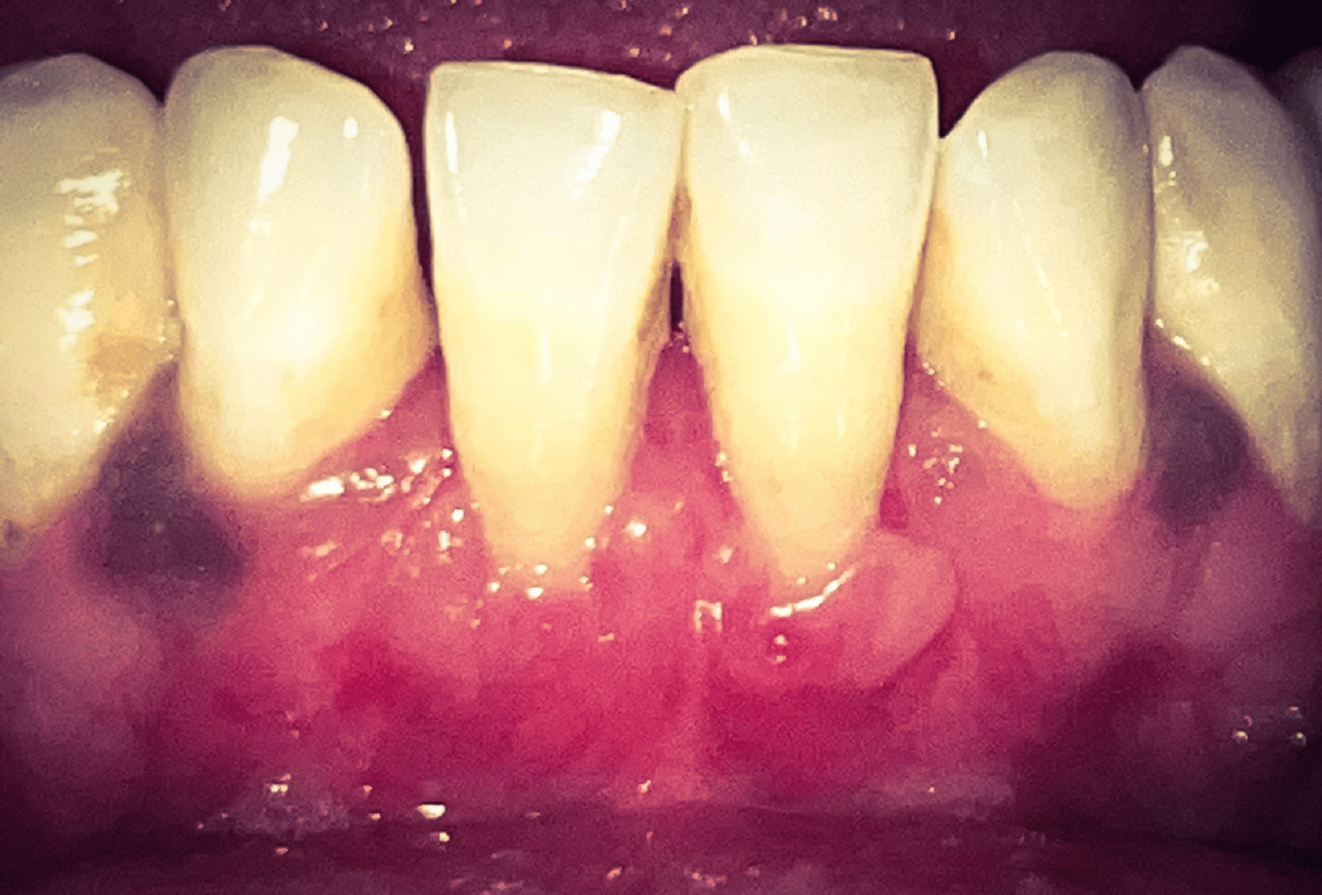
Recipient site at three months postoperatively

At the third month follow-up, the recession depth reduced by approximately 2 mm in relation to both 31 and 41, indicating a significant reduction (Figure 5).

This corresponded to approximately 60% root coverage, along with an increase in the width of keratinized gingiva, improved aesthetic appearance, and a reduction in dentinal hypersensitivity.

## Discussion

The primary objective of periodontal plastic surgery in the treatment of gingival recession is to achieve stable root coverage, increase the width of keratinized gingiva, and enhance aesthetic outcomes. Among the available techniques, the SCTG is widely regarded as the gold standard due to its high predictability and superior aesthetic results [3]. FGG, typically harvested from the palate, is effective in increasing the width of attached gingiva; however, its application in root coverage is limited due to poor color match [4]. DFGG has been introduced to overcome these limitations by combining the advantages of FGG and SCTG, providing improved vascularity, adequate graft thickness, and enhanced aesthetic integration [5]. In the present case, a significant reduction in gingival recession was achieved in relation to 31 and 41 following DFGG placement. Although complete root coverage in Miller’s Class IV defects is unpredictable due to interproximal attachment loss, partial root coverage with meaningful clinical improvement can be obtained, as supported by previous studies [6]. The use of lasers for de-epithelialization represents an important modification. Compared to scalpel techniques, laser-assisted de-epithelialization allows controlled and uniform epithelial removal, improved visibility, and reduced mechanical trauma [7].

Recent studies have demonstrated that laser-assisted de-epithelialization can provide effective epithelial removal with preservation of connective tissue integrity and satisfactory clinical outcomes [8]. Additionally, systematic reviews suggest that laser-assisted approaches offer comparable results to conventional techniques with potential advantages in precision and healing [9]. Furthermore, comparative studies evaluating different de-epithelialization techniques have reported similar clinical outcomes, indicating that the method of epithelial removal may be modified without compromising treatment success [10].

Laser therapy has also been shown to enhance wound healing by stimulating fibroblast proliferation, collagen synthesis, and angiogenesis, thereby improving graft integration [11]. Importantly, recent evidence highlights that palatal donor site healing dynamics and postoperative morbidity significantly influence overall treatment outcomes in grafting procedures [12]. This is particularly relevant in procedures involving free gingival graft harvesting, as performed in the present case. The success of root coverage procedures depends on several factors, including defect characteristics, gingival phenotype, flap design, graft thickness, and vascular supply. The DFGG technique provides a dual blood supply from both the recipient bed and the overlying flap, thereby enhancing graft survival and reducing postoperative shrinkage. However, limitations include technique sensitivity and the requirement for specialized equipment when using a laser. Long-term evidence remains limited, and further randomized controlled trials are necessary to establish the superiority of laser-assisted techniques over conventional approaches.

## Conclusions

Within the limitations of this case report, it can be concluded that the DFGG harvested from the palate is an effective technique for the management of gingival recession. The adjunctive use of a laser for graft de-epithelialization allows precise and controlled removal of the epithelial layer, which may contribute to improved graft handling and favorable healing outcomes. De-epithelialized grafts, which involve removing the surface epithelium, provide better tissue thickness and superior, more natural, and more stable, long-term, cosmetic color matching compared to conventional, non-de-epithelialized grafts. DFGG has demonstrated comparable or better results than traditional SCTG in terms of reducing recession depth and improving clinical parameters. In the present case, a significant reduction in gingival recession, along with an increase in the width of keratinized gingiva and improved aesthetic appearance, was achieved. Although complete root coverage may not be predictable in advanced defects, the technique demonstrated satisfactory clinical results. In the future, well-designed clinical studies with larger sample sizes and long-term follow-up are required to validate the effectiveness and predictability of laser-assisted DFGGs in periodontal plastic surgery.
